# Osteoprotegerin concentration and risk of cardiovascular outcomes in nine general population studies: Literature-based meta-analysis involving 26,442 participants

**DOI:** 10.1371/journal.pone.0183910

**Published:** 2017-08-24

**Authors:** Lena Tschiderer, Johann Willeit, Georg Schett, Stefan Kiechl, Peter Willeit

**Affiliations:** 1 Department of Neurology, Innsbruck Medical University, Innsbruck, Austria; 2 Department of Internal Medicine 3, University of Erlangen-Nuremberg, Erlangen, Germany; 3 Department of Public Health and Primary Care, University of Cambridge, Cambridge, United Kingdom; Shanghai Institute of Hypertension, CHINA

## Abstract

**Background:**

Recent experimental and epidemiological studies have suggested that osteoprotegerin, a key regulator in bone metabolism, may be involved in vascular calcification and atherosclerosis. Our aim was to reliably quantify the associations of osteoprotegerin concentration and incidence of first-ever cardiovascular disease outcomes in the general population.

**Methods:**

Using the electronic databases MEDLINE, EMBASE and Web of Science (January 1975 and April 2017, no language restrictions), nine relevant studies were identified involving a total of 26,442 participants recruited from the general population. Over a mean follow-up of 8.5 years, 2,160 cardiovascular disease, 2,123 coronary heart disease, and 1,102 stroke outcomes were recorded. Study-specific risk ratios were combined with random-effects meta-analysis.

**Results:**

When comparing individuals in the top with those in the bottom third of osteoprotegerin concentration, the combined risk ratio was 1.83 (95% confidence interval: 1.46, 2.30; P<0.001; *I*^2^ = 76.8%) for cardiovascular disease, 1.72 for coronary heart disease (1.26, 2.37; P = 0.001; *I*^2^ = 83.5%), and 1.58 for stroke (1.18, 2.12; P = 0.002; *I*^2^ = 65.2%). Associations appeared stronger at younger age (P = 0.018 for cardiovascular disease), in studies that did not employ statistical adjustment (P = 0.023 for cardiovascular disease and 0.018 for coronary heart disease), and potentially in studies that measured osteoprotegerin in plasma rather than in serum (P = 0.005 for cardiovascular disease and 0.018 for coronary heart disease). Magnitudes of associations did not differ according to the proportion of males, geographical region, or osteoprotegerin assay manufacturer. There was no evidence for publication bias for any of the outcomes assessed (all P>0.05).

**Conclusions:**

Elevated osteoprotegerin concentration is associated with an increased risk of incident cardiovascular disease in the general population. The mechanisms underlying this observation deserve further investigation.

## Introduction

Osteoprotegerin (OPG) was initially discovered in 1997 as a key regulator in bone metabolism [1] and has meanwhile been implicated in various human diseases including myocardial infarction, stroke, aortic aneurysm, atrial fibrillation, aortic stenosis, chronic kidney disease, diabetes and its complications, and cancer [2–4]. OPG is expressed in normal vasculature, but several-fold unregulated in the failing myocardium, abdominal aortic aneurysm specimens, and advanced complicated atherosclerotic plaques [2,5–7]. In arterial tissue it acts as a calcification inhibitor, counteracts plaque destabilizing effects of RANKL, and exerts various direct both favourable and unfavourable effects [2,8–15]. OPG in circulation partly originates from vascular tissue [16] making it a promising vascular biomarker.

A considerable number of population-based studies have analysed the association between circulating OPG concentration and onset of cardiovascular diseases (CVD). These studies reported associations of varying magnitudes and employed different levels of adjustments. Comparability and interpretation of the reported effect sizes have been complicated by differing scales across studies (i.e. reported effect sizes per unit higher OPG vs. per SD higher OPG vs. quantile comparisons). Previous attempts to provide a systematic overview on the associations on OPG and CVD outcomes allowed for retrospective studies and/or had a focus on high-risk individuals [17–19].

The aim of the present meta-analysis was three-fold. First, to identify and collate all available studies reporting on OPG concentration and cardiovascular risk in the general population. Second, to calculate a combined relative risk for the association of OPG with a composite cardiovascular disease (CVD) endpoint and its components coronary heart disease (CHD) and stroke by use of meta-analytical techniques. Third, to explore whether certain study characteristics, such as geographical region or analytic characteristics (e.g. sample type) have an impact on the magnitudes of these associations.

## Methods

### Literature search, study selection, and data extraction

We searched the literature for prospective studies that had been published between January 1970 and April 2017 and reported on associations of OPG concentration with a composite CVD endpoint, CHD (defined as non-fatal myocardial infarction or coronary death), or stroke. Systematic searches of PubMed, Web of Science and EMBASE were supplemented by scanning reference lists of articles identified (including reviews) and by correspondence with several study investigators. The search strategy did not apply language restrictions; search terms are detailed in Table 1.

**Table 1:** Search terms used to identify relevant articles.

PubMed
("OPG" [All Fields] OR "Osteoprotegerin" [All Fields] OR "OCIF Protein" [All Fields] OR "Osteoclastogenesis Inhibitory Factor" [All Fields] OR "Tumor Necrosis Factor Receptor 11b" [All Fields] OR "Osteoprotegerin"[Mesh]) AND ("Cardiovascular Diseases" [Mesh] OR "Coronary Artery Disease" [MeSH] OR "Atherosclerosis" [MeSH] OR "Coronary Disease" [MeSH] OR "Myocardial Infarction" [MeSh] OR "Myocardial Ischemia" [MeSH] OR "Stroke" [MeSH] OR "Cerebrovascular" [All fields]) NOT ("Animals"[MeSH] NOT "Humans"[MeSH])
Web of Science
TS = ("OPG" OR "Osteoprotegerin" OR "OCIF Protein" OR "Osteoclastogenesis Inhibitory Factor" OR "Tumor Necrosis Factor Receptor 11b") AND TS = ("Cardiovascular Diseases" OR "Coronary Artery Disease" OR "Atherosclerosis" OR "Coronary Disease" OR "Myocardial Infarction" OR "Myocardial Ischemia" OR "Stroke" OR "Cerebrovascular")
EMBASE
("OPG" OR "Osteoprotegerin" OR "OCIF Protein" OR "Osteoclastogenesis Inhibitory Factor" OR "Tumor Necrosis Factor Receptor 11b").af AND ("Cardiovascular Diseases" OR "Coronary Artery Disease" OR "Atherosclerosis" OR "Coronary Disease" OR "Myocardial Infarction" OR "Myocardial Ischemia" OR "Stroke" OR "Cerebrovascular").af

No language restrictions were applied.

Studies were eligible for inclusion if they had met all of the following criteria: (1) had recruited study participants from the general population (i.e. selected participants not on the basis of having preexisting disease at baseline); (2) were prospective in design; and (3) had a follow-up for incident cardiovascular outcomes of more than 1 year.

Using standardized data extraction protocols, two independent reviewers (L.T., P.W.) extracted–by consensus–information on geographical location, baseline survey dates, study design, duration of follow-up, mean age at baseline, proportion of male participants, sample type, OPG assay manufacturer, total number of participants in the study, the number of incident CVD, CHD and stroke outcomes, effect sizes, and the degrees of statistical adjustment of reported associations. Hazard ratios and odds ratios were assumed to approximate the same measure of relative risk (RR). When a study had reported effect estimates for different levels of adjustment, we extracted the estimate relating to the most fully adjusted model. The degree of adjustment was classified as: ‘o’ when RRs were unadjusted; ‘+’ when RRs were adjusted for age and sex only; and ‘++’ when RRs were additionally adjusted for at least one non-blood based risk factor (e.g. smoking, history of diabetes, blood pressure) and one blood-based risk factor (e.g. total cholesterol, HDL cholesterol, C-reactive protein, NT-proBNP). The quality of each included study was assessed using the Newcastle-Ottawa Scale for cohort studies [20], which assesses participant selection, exposure measurement, outcome ascertainment, covariate adjustment and follow-up, and can range from zero points (low quality) to nine points (high quality). If multiple publications on the same study were available, the most up-to-date or comprehensive information was used. The meta-analysis was conducted following the PRISMA guidelines (the PRISMA checklist is available as supporting information, see S1 Table) [21].

### Statistical analyses

We conducted the statistical analysis according to a pre-defined statistical analysis plan. The combined CVD endpoint was defined as CHD and stroke. Study-specific risk estimates were pooled using random-effects meta-analyses (with sensitivity analyses using fixed-effect meta-analyses). To enable a consistent approach to analysis, RRs and 95% confidence intervals in each study were standardized to a common scale, i.e. to reflect a comparison of the top third with the bottom third of the population's OPG distribution, employing statistical methods described elsewhere [22]. Consistency of findings across studies was assessed with standard χ^2^ tests and the *I*^2^ statistic [23]. Subgroup analyses were conducted using meta‐regression across pre‐specified study‐level characteristics [23]. Evidence of publication bias was assessed using funnel plots and Egger's asymmetry test [24]. Statistical tests were 2‐sided and used a significance level of P<0.05.

## Results

### General characteristics of included studies

**Fig 1:**
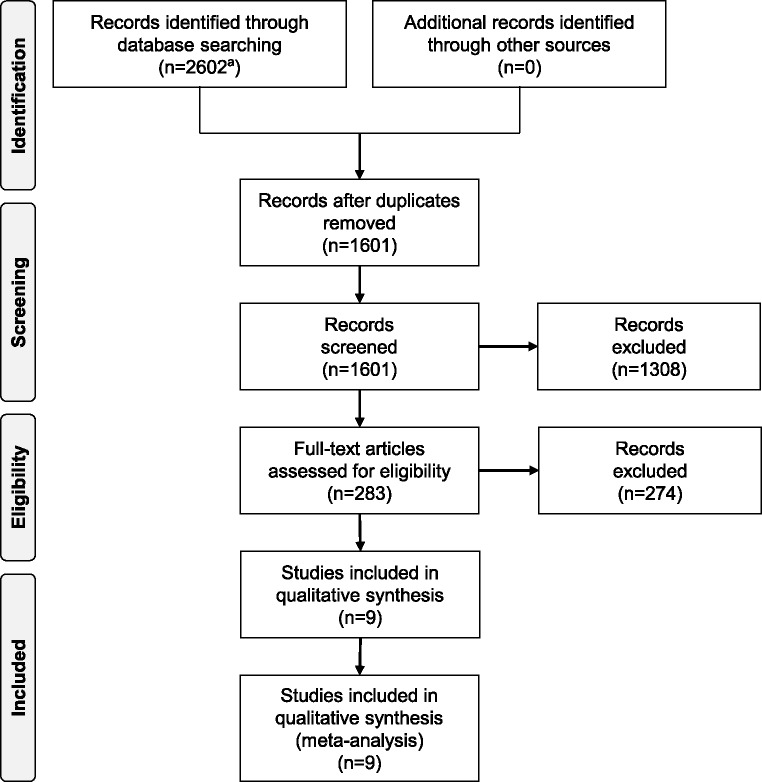
PRISMA flow diagram. ^a^We identified 823 records in Pubmed, 728 in Web of Science, and 1052 in EMBASE.

We screened 2,602 records (Fig 1) and identified nine eligible prospective studies [25–33] reporting on a total of 26,442 participants (Table 2). Baseline survey years of the eligible studies ranged from 1982 to 2011. Five were based in Europe, two in the USA, one in Asia, and one in Australia. The quality of the studies was high, with a mean Newcastle-Ottawa Scale score of 7.3. Over a weighted mean follow-up duration of 8.5 years, a total of 2,160 CVD, 2,123 CHD, and 1,102 stroke outcomes were recorded. The majority of studies used ELISA-based methods to quantify circulation OPG concentration. One study reported unadjusted effect estimates only [29], two studies reported estimates adjusted for age and sex [25,32], and six reported further adjusted estimates [26–28,30,31,33]. On average, participants were 60.5 years old at baseline and 43.5% were male.

**Table 2:** Characteristics of nine prospective population-based studies included in the meta-analysis.

Study	Location	Year of baseline survey, range	Study design	Study quality, NOS score	Maximum follow-up, years	Mean age, years	Male sex, %	OPG assay type (manufacturer)	Sample type	No. of participants	No. of events		
											CVD	CHD	Stroke
Browner et al. [25]	USA	1986–88	NCC	5	11.5	72.0	0.0	ELISA (Amgen)	Serum	490	51^d^	-	241
BRUN [26,34]	Italy	1990	PC	8	10.0	58.8	50.4	ELISA (Biomedica)	Serum	909	124	63	66
CAIFOS [27]	Australia	1998	PC	8	15.0	75.2	0.0	ELISA (R&D Systems)	Serum	1,292	210^d^	-	-
CCHS [29]	Denmark	2001–03	PC	9	9.7	59.0	42.6	IFMA (R&D Systems)	Plasma	5,863	616	462	154
DCH [30]	Denmark	1993–97	NCC	6	6.0	60.5^b^	61.0	ELISA (R&D Systems)	Plasma	508	-	-	254
EPIC-Norfolk [31]	UK	1993–97	NCC	6	6.7^a^	65.0	63.7	ELISA (R&D Systems)	Serum	2,656	-	951	-
FRAM-OC [28]	USA	1998–01	PC	8	4.6^a^	61.0	46.0	ELISA (Biomedica)	Serum	2,933	132	72	53
Shen et al. [32]	China	2003	PC	7	5.0	55.9	40.9	NR	Plasma	6,092	118	-	-
Tromso [33]	Norway	1994–95	PC	9	12.0	25–85^c^	47.4	ELISA (R&D Systems)	Serum	5,699	909	575	334
TOTAL		1982–11		7.3	8.5	60.5	43.5			26,442	2,160	2,123	1,102

Abbreviations: BRUN = Bruneck Study; CAIFOS = Calcium Intake Fracture Outcome study; CCHS = Copenhagen City Heart Study; DCH = Diet, Cancer, and Health; ELISA = enzyme-linked immunosorbent assay; EPIC-Norfolk = European Prospective Investigation into Cancer and Nutrition—Norfolk; FRAM-OC = Framingham Study—Offspring Cohort; IFMA = immunofluoremetric assay; NCC = nested case-control study; NOS, Newcastle-Ottawa Scale; NR = not reported; OPG = osteoprotegerin; PC = prospective cohort study; Tromso = The Tromso Study.

Summary statistics are ranges, weighted means, or sums, as appropriate.

^a^mean

^b^median

^c^range.

^d^fatal CVD

### Overall associations of OPG with cardiovascular outcomes

**Fig 2:**
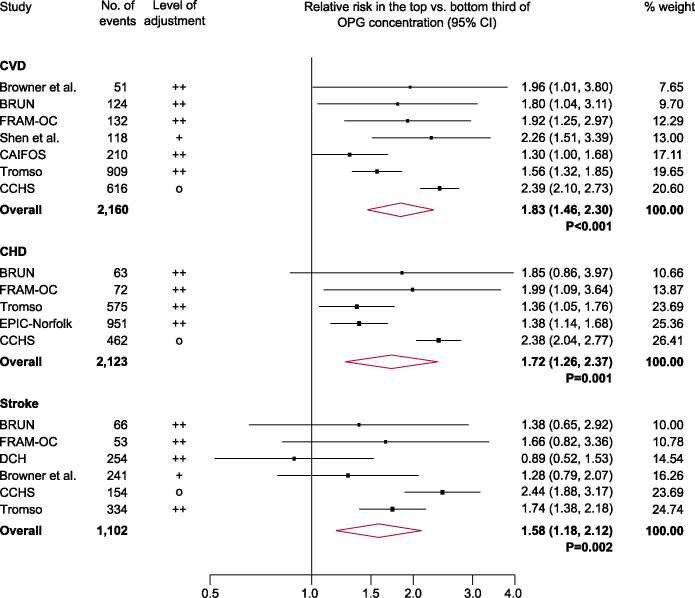
Combined relative risk for cardiovascular outcomes in the top vs. the bottom third of OPG concentration. CHD = coronary heart disease; CI = confidence interval; CVD = cardiovascular disease; OPG = osteoprotegerin. Full study names are listed in the footnote of Table 2. Sizes of data markers indicate the weight of each study in the analysis. Study-specific relative risks were pooled using random-effects meta-analysis.

**Fig 3:**
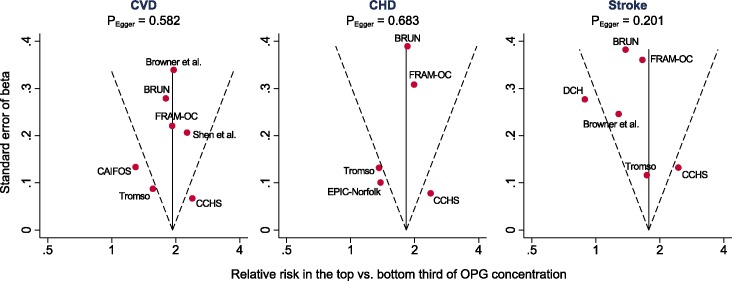
Funnel plots of reported associations between osteoprotegerin and risk of cardiovascular outcomes. Full study names are listed in the footnote of Table 2. The dotted lines show pseudo 95% confidence intervals around the overall pooled estimate. P values are from Egger’s asymmetry test of associations.

A forest plot of associations of OPG with cardiovascular outcomes is provided in Fig 2. The combined relative risk for CVD was 1.83 (95% CI: 1.46, 2.30; P<0.001) for a comparison of individuals in the top with those in the bottom third of baseline values of OPG concentration. The corresponding relative risks for CHD and stroke were 1.72 (1.26, 2.37; P = 0.001) and 1.58 (1.18, 2.12; P = 0.002), respectively. The degree of between-study heterogeneity was high with *I*^2^ values of 76.8% for CVD (P<0.001), 83.5% for CHD (P<0.001), and 65.2% for stroke (P = 0.014). Sensitivity analyses that used fixed-effect meta-analyses (rather than random-effects meta-analysis as in the primary analysis) yielded relative risks of 1.93 (1.77, 2.11) for CVD, 1.83 (1.64, 2.03) for CHD and 1.77 (1.53, 2.06) for stroke. There was no evidence for publication bias, as illustrated by funnel plots and tested with Egger’s asymmetry test (Fig 3).

In a subset of three studies with concomitant information on OPG and RANKL [26,28,32], the association with CVD was stronger for CVD than for RANKL, with relative risks of 2.12 (1.64, 2.74) and 1.34 (1.06, 1.68) for a top vs. bottom third comparison.

### Findings according to study characteristics

**Fig 4:**
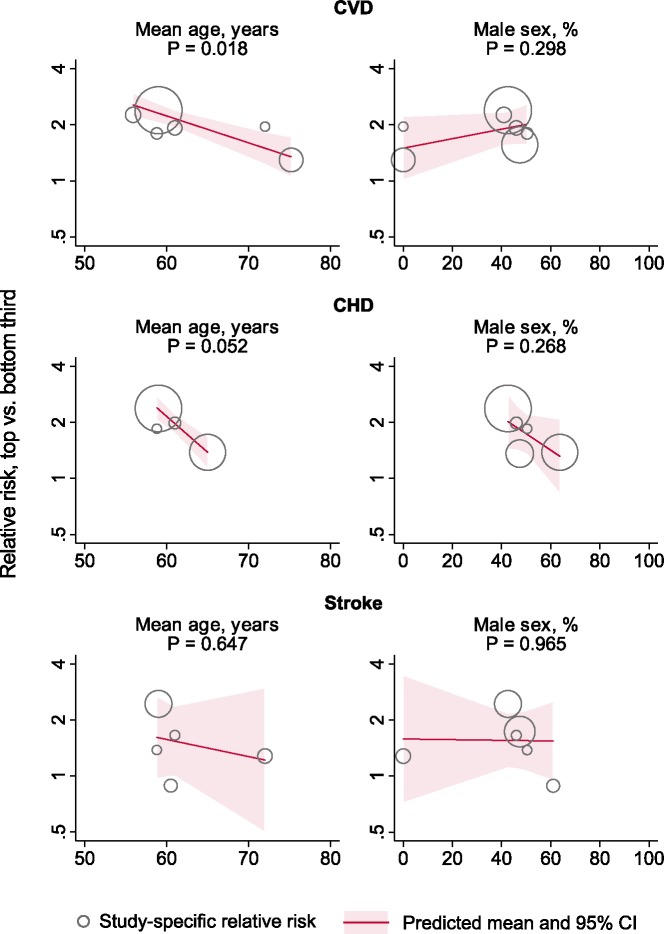
Relative risks for cardiovascular outcomes in the top vs bottom third of osteoprotegerin concentration according to average age and proportion of males in contributing studies. P values were derived from meta-regression. The marker size is proportionate to the inverse variance of the study-specific relative risk.

**Fig 5:**
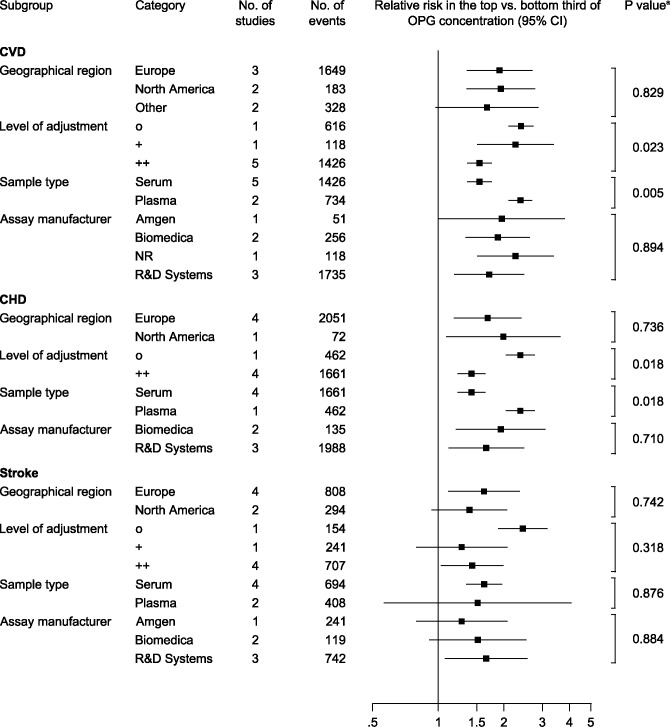
Relative risks for cardiovascular outcomes in the top vs bottom third of osteoprotegerin concentration according to categories of study characteristics. ^a^P values were derived from meta-regression. Levels of adjustment: o, unadjusted; +, adjusted for age and sex; ++, additionally adjusted for at least one non-blood based risk factor and one blood-based risk factor.

Fig 4 and Fig 5 compare the magnitudes of associations between OPG and cardiovascular outcomes according to several study characteristics. The association between OPG and CVD risk was weaker at older age (P = 0.018), but did not differ according to the proportion of males included in the studies (Fig 4). We noted a stronger association for studies reporting unadjusted estimates for the CVD (P = 0.023) and the CHD outcome (P = 0.018) (Fig 5). Furthermore, for the outcomes CVD and CHD, there was evidence for stronger associations in the two studies that measured OPG in plasma [27,29] than in the other studies that had analysed serum (P = 0.005 and 0.018). One of these two studies [29], however, reported only unadjusted associations, which may have led to an overestimation of serum/plasma effects. Exclusion of this study in a sensitivity analysis increased the P value for CVD to 0.154. There was no evidence for a difference in the magnitude of associations according to geographical region or OPG assay.

## Discussion

The present meta-analysis included about 26,000 participants from nine prospective cohort studies across seven countries. Overall, compared with individuals in the bottom third of baseline OPG concentration, those in the top third of baseline OPG concentration were at a 83% higher risk of CVD.

The mechanistic underpinning and nature of the association between OPG and CVD is complex. First, multiple associations have been reported to exist between circulating OPG and cardiovascular risk factors (including age, smoking, hypertension, insulin resistance, obesity, diabetes, and renal impairment) as well as inflammatory diseases with heightened CVD risk, such as inflammatory bowel disease [2–4]. Intriguingly, amelioration of risk factor profile or treatment of inflammation results in a lowering of OPG levels [3,4,35,36]. Accordingly, OPG reflects a cumulative exposure to and burden of known and emerging vascular risk factors. However, the association between OPG and CVD maintains significance even after careful control for all these risk factors, which suggests an additional mechanistic role for OPG. In this context, OPG level is assumed to be a reliable indicator of the overall activity of the OPG/RANK/RANKL system. OPG is a soluble decoy receptor for RANKL and TRAIL, thereby inhibiting ligation of these mediators with the cognate receptors and blocking subsequent pro-inflammatory, calcifying, and pro-apoptotic consequences (beneficial effects) [8]. On the other hand, accumulating evidence supports the concept that sustained prominent upregulation of OPG provokes detrimental pro-fibrotic, pro-inflammatory (e.g. facilitation of monocyte chemotaxis) and paradoxically also pro-apoptotic (smooth muscle cells) and plaque destabilizing MMP-releasing effects for OPG [10–15]. Accordingly, under certain circumstances, OPG may also act as a risk factor for CVD. The potential role in plaque destabilization has been underpinned by upregulation in unstable angina compared to stable angina [11], and in unstable compared to stable carotid atherosclerosis tissue [7]. While the OPG/RANK/RANKL system is complex and tightly intertwined [37], our sensitivity analysis in three studies showed markedly stronger associations with CVD for OPG than for RANKL. In a recent meta-analysis of genetic association studies, carriers of polymorphisms in the OPG gene (rs2073617 and rs2073618) linked to higher circulating OPG levels faced an increased risk of CVD, but this only referred to Asians and not to Caucasians [38]. As a potential explanation, heritability of OPG level was reported to be as high as 75% in Chinese women, but only 23% and 24% in predominantly white female and male participants of the Framingham Study [39].

From a biomarker perspective, OPG is appealing in view of very low intra-individual variability overtime (five-year correlation coefficient r = 0.71) [26], easy measurement by commercially available ELISAs (measuring the composite of OPG monomer, OPG dimer, and OPG bound to RANKL and TRAIL) and standards, high stability under usual storage conditions (more than ten years at -70°C) and comparatively low pre-analytic variability [40]. However, OPG concentration was shown to rise if sample processing is delayed by 6 hours or more, centrifuged serum samples are stored for more than 48 hours at room temperature, or frozen samples undergo four or more freeze-thawing cycles [40]. Moreover, heparin treatment temporarily doubles OPG levels (for about 1 hour) probably by prominent OPG release from vascular smooth muscle cells [41,42]. OPG concentrations are somewhat higher in plasma samples [40,43] and our primary analysis suggested a somewhat stronger association of plasma OPG. However, because this was not confirmed in a sensitivity analysis that omitted a study that did not employ statistical adjustment [29], the relative predictive utility of OPG measured in different blood specimen remains to be determined. Finally, with a risk ratio of 1.83 (1.46, 2.30) for CVD for a comparison of extreme OPG thirds, the association we observed was strong. For example, previous literature-based meta-analyses on other emerging biomarkers yielded risk ratios of 2.82 (2.40, 3.33) for B-type natriuretic peptides [44], 1.42 (1.29, 1.56) for asymmetric dimethylarginine [45], 1.40 (1.15, 1.70) for leukocyte telomere length [46].

There are a number of strengths and weaknesses of the current study that merit discussion. The meta-analysis combined large-scale data from >26,000 participants and >2,100 CVD events and was thereby able to provide more precise estimates of association than any single study before. Transformation of study-specific HRs to reflect relative risk between those with OPG values in the upper third compared with lower third allows a common scale to be used when pooling studies and has been successfully applied in the assessment of a number of biomarkers [22]. It does, however, assume that the relationship between the biomarker of interest and disease risk is linear. Actually, several of the individual studies have suggested a dose-response relationship for OPG and cardiovascular outcomes [26,31,33]. In the meta-analysis, there was significant between-study heterogeneity which could have resulted from a number of differences between the studies included as discussed above. There is emerging evidence on associations of OPG with heart failure [47,48] and left ventricular structure and function [49], but studies were too few to be included in the present meta-analysis. Inherent limitations exist in the use of study-level data. Availability of individual-participant data would allow for further standardization of analytical techniques and covariate adjustment and more comprehensive subgroup analysis.

## Conclusion

Elevated OPG concentration is associated with an increased risk of incident cardiovascular outcomes in the general population. The mechanism underlying this observation deserves further investigation as does the predictive performance of OPG as a biomarker in clinical routine.

## Supporting information

S1 Table PRISMA checklist. (PDF)

S1 File Data extracted for the meta-analysis. (XLSX)
